# SCOPE: safer care for older persons (in residential) environments—a pilot study to enhance care aide-led quality improvement in nursing homes

**DOI:** 10.1186/s40814-022-00975-8

**Published:** 2022-02-03

**Authors:** Malcolm Doupe, Thekla Brunkert, Adrian Wagg, Liane Ginsburg, Peter Norton, Whitney Berta, Jennifer Knopp-Sihota, Carole Estabrooks

**Affiliations:** 1 Max Rady College of Medicine, Rady Faculty of Health Sciences, University of Manitoba, Winnipeg, MB Canada; 2 University Department of Geriatric Medicine FELIX PLATTER, Basel, Switzerland; 3 Nursing Science (INS), Department Public Health (DPH), Faculty of Medicine, University of Basel, Basel, Switzerland; 4 Department of Medicine, Faculty of Medicine and Dentistry, University of Alberta, Edmonton, Alberta Canada; 5 School of Health Policy & Management, York University, Toronto, Canada; 6 Department of Family Medicine, University of Calgary, Calgary, Alberta Canada; 7 Institute of Health Policy, Management & Evaluation, University of Toronto, Toronto, Canada; 8 Faculty of Health Disciplines, Athabasca University, Edmonton, Alberta Canada; 9 Faculty of Nursing, University of Alberta, Edmonton, Alberta Canada

**Keywords:** Nursing homes, Quality improvement, Pilot study, Care aide-led intervention, Facilitated coaching, Enactment

## Abstract

**Background:**

Nursing home residents require daily support. While care aides provide most of this support they are rarely empowered to lead quality improvement (QI) initiatives. Researchers have shown that care aide-led teams can successfully participate in a QI intervention called Safer Care for Older Persons in Residential Care Environments (SCOPE). In preparation for a large-scale study, we conducted a 1-year pilot to evaluate how well coaching strategies helped teams to enact this intervention. Secondarily, we measured if improvements in team cohesion and communication, and resident quality of care, occurred.

**Methods:**

This study was conducted using a prospective single-arm study design, on 7 nursing homes in Winnipeg Manitoba belonging to the Translating Research in Elder Care research program. One QI team was selected per site, led by care aides who partnered with other front-line staff. Each team received facilitated coaching to enact SCOPE during three learning sessions, and additional support from quality advisors between these sessions. Researchers developed a rubric to evaluate how well teams enacted their interventions (i.e., created actionable aim statements, implemented interventions using plan-do-study-act cycles, and used measurement to guide decision-making). Team cohesion and communication were measured using surveys, and changes in unit-level quality indicators were measured using Resident Assessment Instrument-Minimum Data Set data.

**Results:**

Most teams successfully enacted their interventions. Five of 7 teams created adequate-to-excellent aim statements. While 6 of 7 teams successfully implemented plan-do-study-act cycles, only 2 reported spreading their change ideas to other residents and staff on their unit. Three of 7 teams explicitly stated how measurement was used to guide intervention decisions. Teams scored high in cohesion and communication at baseline, and hence improved minimally. Indicators of resident quality care improved in 4 nursing home units; teams at 3 of these sites were scored as ‘excellent’ in two or more enactment areas, versus 1 of the 3 remaining teams.

**Conclusions:**

Our coaching strategies helped most care aide-led teams to enact SCOPE. Coaching modifications are needed to help teams more effectively use measurement. Refinements to our evaluation rubric are also recommended.

**Supplementary Information:**

The online version contains supplementary material available at 10.1186/s40814-022-00975-8.

## Key messages regarding feasibility

This pilot provides knowledge to guide future care aide-led nursing home quality improvement initiatives, by:

- Showing that care aides can effectively lead these initiatives (e.g., enact Plan-Do-Study-Act principles that in some instances resulted in improved quality of resident care);
- Demonstrating that PDSA training to support these initiatives should emphasize the interconnected nature of AIM development, care plan implementation, and measurement;
- Providing insights into ways in which the SCOPE intervention could be further modified (e.g., by using less didactic teaching, providing teams with practical measurement tools), and;
- Showing how a (draft) rubric can measure fidelity enactment and suggesting approaches to refine this tool.

## Background

Older adults are the fastest growing segment of the worldwide population [1]. As life expectancy increases so does the number of people with dementia and other co-morbid medical conditions [2–6]. Similarly, the care needs of nursing home residents have also increased substantially in recent years [7]. Annually, 1.7 million North Americans reside in nursing homes [8], and at least half of these residents have some form of age-related dementia often combined with additional impairments such as difficulties completing daily tasks, responsive behaviours, and frequent incontinence [9–11]. This vulnerable group requires complex health, personal, and social care, provided in ways that has meaning for residents [12] and that emphasize the importance of relational care and quality of life [13]. While media have highlighted the significant challenges with nursing home care during pandemic times [14–16], the quality of care provided in this sector has been recognized as suboptimal for decades, and many groups have called to improve nursing home structures and care processes [17–21].

Care aides (unregulated workers, also called personal support workers, orderlies or nursing assistants) provide 80–90% of direct care to nursing home residents in Canada [22]. These staff are best situated to observe, interpret, and respond to residents’ daily needs [23, 24], making them uniquely positioned to meaningfully participate in and, we contend, to lead quality improvement (QI) initiatives. These staff often have little formal vocational training, frequently speak English as a second language, yet conduct a wide range of care activities that are unregulated by any professional organization [25, 26]. Despite their important role, care aides are rarely included in formal care planning processes, making them feel under-valued by other care staff and emphasizing the need to create more constructive collaborative care approaches [27]. Evidence shows that empowering care aides enhances their work performance and quality of work life [28–30], and that improving inter-professional collaboration can enhance the quality of nursing home care [31, 32].

Given this knowledge, we previously developed an intervention called Safer Care for Older Persons in Residential Care Environments (SCOPE) [33]. SCOPE is a multi-component intervention designed to empower care aides to lead, with coaching support, QI activities that help them to use best evidence in their practice, and secondarily to improve their quality of work life and engagement. Enhancements in these areas should ultimately lead to improved quality of resident care and their associated health-related outcomes. In a previous publication, researchers have shown that care aides (1) have great interest and are willing to actively participate in SCOPE (e.g., by attending learning sessions and submitting intervention progress reports), and (2) are able to apply SCOPE principles at the resident bedside and hence contribute to quality care improvement [34].

Based on these findings and in preparation for a larger trial, we further developed and operationalized SCOPE facilitated coaching strategies, created a rubric to measure how well teams were able to implement this initiative, and piloted the revised intervention for 1 year. The primary aim of this manuscript is to describe how well our revised coaching strategies helped teams to enact their QI interventions (i.e., to create actionable QI aim statements, implement their QI plans using plan-do-study-act [PDSA] cycles, and use measurement to guide decisions about the need to modify their intervention approaches). As a secondary aim, we also used surveys to measure improvement in team cohesion and communication during SCOPE, and used Resident Assessment Instrument-Minimum Data Set (RAI-MDS 2.0) data to describe changes in select quality indicators at the resident care unit-level.

## Methods

### Study design

This was a single arm prospective pilot study, lasting 1 year from 8 February 2016 to 10 February 2017.

### Ethics

Approval to conduct the research was provided by the University of Manitoba Health Research Ethics Committee (reference number H2015:045). This study was funded by the TREC program (grant number PS 148582). Participating homes received $3000 to offset the costs of participation such as backfilling staff who attended learning sessions.

### The translating research in elder care research program

Translating Research in Elder Care (TREC) is a multilevel, longitudinal program of applied health services research designed to improve the quality of care and quality of life for nursing home residents, and also the quality of work life for their care staff [35]. TREC applies these constructs at the clinical microsystem (care units) where quality is created [36, 37]. The overall TREC cohort was created using a random sample across three Canadian provinces (Alberta, Manitoba, British Columbia), stratified by owner-operator type and size [35]. TREC data are provided on about 94 Canadian nursing homes comprised of 334 units; 5500 care aides, nurses, and other care providers; and 31000 RAI-MDS 2.0 assessments completed on 13,800 residents. Nursing homes for this pilot were selected from the Manitoba TREC cohort as described below in the Participants and Study Procedures section.

### The SCOPE teaching and coaching strategies

**Fig. 1:**
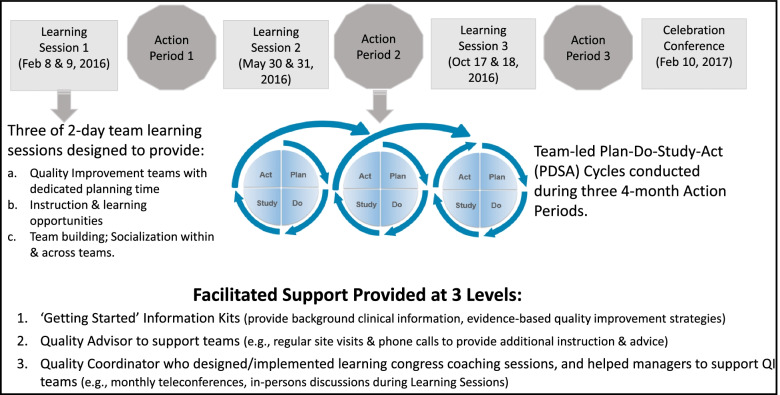
Safer Care for Older Persons in Residential Care Environments (SCOPE) pilot study coaching strategies (February, 2016–February, 2017)

The SCOPE intervention is based on a modified *Institute for Healthcare Improvement (IHI) Breakthrough Collaborative Series* model [38]. This model uses the Plan-Do-Study-Act (PDSA) approach to improving care that teaches organizations to formally develop AIM statements, to iteratively test change ideas on small groups before more fully implementing them, and to use measurement to evaluate change [38]. SCOPE is also informed by knowledge translation theory, specifically focusing on the important role that facilitation plays in implementation projects [39, 40]. Each component of the SCOPE coaching strategy is shown in Fig. 1, with further details provided elsewhere [33, 41]. These include the following:

- ‘Getting Started’ evidence kits that provide (topic-specific) background clinical information and evidence-informed ideas for improving care;
- Three 2-day learning sessions (i.e., workshops attended by all teams) designed to train teams about PDSA quality improvement approaches, and to provide them with peer networking and learning opportunities;
- A quality advisor who helped to design and implement the learning session, and who supported teams using in-person visits and telephone calls regularly between learning sessions;
- A quality coordinator who led virtual and in-person discussions to help unit and facility managers support front-line QI teams, and supported the quality advisor when needed, and;
- A celebratory conference held at the end of the pilot.

The quality advisor was the main liaison with each team. Duties included the following:

- Meeting with each team at the beginning of SCOPE to review the ‘Getting Started’ information kit;
- Working with the quality coordinator and research team to prepare and facilitate learning sessions;
- Conducting face-to-face meetings with each team at least monthly, to help them enact their PDSA plans and brainstorm solutions to challenges encountered;
- Being available for additional team consultation as needed; and,
- Keeping a diary of team interactions and progress.

Learning sessions occurred 3 months apart (Fig. 1); the agenda for each session is provided in Appendix 1. In learning session 1, teams were coached to develop effective QI aim statements, while learning sessions 2 and 3 focused on measurement and strategies to spread effective QI strategies within each team’s unit, respectively. Coaching strategies used in the learning sessions included improv and simulation techniques, and interactive “games” designed to promote PDSA training. Time was also dedicated to help teams problem solve and share solutions to challenges that they encountered (e.g., getting buy-in from peers), to provide teams with knowledge sharing and socialization opportunities (e.g., networking sessions and team presentations sharing their PDSA experiences), and to discuss how lessons learned could be integrated into daily care routines. During the final celebratory conference, teams celebrated their achievements, discussed lessons learned, and considered next steps.

SCOPE pilot nursing homes focused their change ideas on one of three clinical areas (reducing pain, improving mobility, and reducing dementia-related responsive behaviours). As explained by Cranley et al. (2011), these areas were selected using a Delphi method applied to gerontology experts; senior decision-makers; and nursing home care aides, registered nurses, and care managers/educators [33]. At the onset of SCOPE, TREC senior decision-making partners requested that we replace one of the original priority areas (prevention and management of pressure ulcers) with responsive behaviors, to reflect clinical areas that needed improvement and where change strategies could feasibly be identified and implemented by care aides. We limited the number of clinical options offered in the pilot, to help optimize sharing and learning among teams working on the same target areas.

### Participants and study procedures

Nursing homes were randomly selected from within the Manitoba TREC cohort by one of the co-authors (PN) using a random number table without replacement. This process was stratified by owner-operator type (voluntary not for profit, public not for profit, private for profit) and facility size (small, medium, and large), ensuring that the number of sites selected in each stratum were proportional to the overall TREC cohort. While we had originally planned to recruit 8 sites, one site declined to participate stating insufficient staff levels to engage in research. No sites were lost to follow-up during this pilot.

Executive Directors from each facility received a written invitation to participate in the pilot followed by an in-person meeting to answer questions, to explain nursing home responsibilities, and to discuss available support. Following written consent to participate in the pilot, the Executive Director identified a senior sponsor (usually the Director of Care) to help promote SCOPE to other management staff, and to remove implementation barriers throughout the pilot as needed. This latter individual identified, at their discretion, one unit from their facility to participate in the pilot, and selected a unit-level team sponsor (usually a unit-level clinical nurse manager) who was responsible for supporting day-to-day project activities. Senior and Team Sponsors collaborated to select a front-line team consisting of 5–7 members. At least 2 team members were care aides with one as team lead; other care staff (e.g., social workers) were selected as needed. Sites used various strategies (decided by sponsors, team consensus based on resident need) to select one of the three clinical areas to work on.

## Measures and data analysis

### Treatment enactment

Enactment is an element of treatment fidelity that measures the extent to which people actually implement an intervention and differs from what is taught (treatment delivery), what is learned (treatment receipt), and the extent of its effect (treatment efficacy) [42]. Enactment is one of the most challenging aspects of treatment fidelity to measure [42, 43]. Traditional approaches to measuring it include the use of questionnaires and self-reports, structured interviews, observation, and activity logs [42].

Each team was asked to self-report their implementation progress every 2 months during the pilot, using a PDSA progress worksheet (Appendix 2). Teams used this worksheet to document (1) refinements made to their QI aim statement; (2) how well they were able to implement QI interventions using PDSA methods (e.g., starting with one or two residents, and involving other residents and/or staff depending on their success); and (3) the extent to which they used data and measurement strategies to guide team decision-making.

Researchers developed a rubric to measure “enactment” in each of these areas, based on the information that teams provided, and using a 5-point scale ranging from poor (1) to excellent (5). As one example, aim statements were scored by the extent that teams met the SMART criteria of being Specific, Measurable, Achievable, Relevant, and Timely [44]. Detailed criteria and scoring definitions for each area of enactment are provided in Table 1. Two authors (MD, LG) independently reviewed each team’s self-reported responses as documented using the PDSA progress worksheets, and provided a team score for each enactment area. Scoring discrepancies were resolved through iterative discussions.

**Table 1:** Scoring system used to rate team’s level of treatment enactment during SCOPE

Treatment enactment category	Scoring based on teams’ self-reported progression throughout the pilot		
	Excellent (5)	Adequate (3)	Poor (1)
**Creating actionable AIM statements**^**a**^	The team developed an aim statement that reflects 4 of 5 of the SMART components including the ‘specific’ and ‘measurable’ categories.	The team developed an aim statement that reflects up to 3 of the SMART components.	The team’s aim statement did not reflect any of the SMART components.
**Intervention Progression using Plan-Do-Study-Act (PDSA Cycles**	The team planned and implemented their intervention in a way that aligned with their aim statement, AND reported using PDSA cycles to spread it to involve other residents and/or staff on their unit.	The team planned and implemented their intervention, but it did not align clearly with their aim statement, OR was only conducted on a limited number of residents and/or staff on the unit.	The team provided no evidence of implementing their intervention, or using PDSA cycles to promote change
**Use of measurement to guide decision-making**	The team included specific text documenting how measurement and data were used to guide improvement decisions in successive PDSA cycles.	The team made vague reference to measurement tools and/or strategies used to guide decision-making in successive PDSA cycles.	The team did not report how measurement and data were used to guide decision-making.

^a^Team aim statements had to include operational terms (e.g., define responsive behavior) (*Specific*); contain a target goal (e.g., identify the degree of improvement sought) (*Measurable*); be realistic (e.g., initially focus on a smaller number of residents) and/or show progression throughout the pilot (*Achievable*); include information about how (e.g., by creating toolkits to support implementation) or when (e.g., during mealtime) the intervention would happen (*Relevant*), and; include a reference point/date by which intervention success would be measured (*Timely*)

### Workgroup cohesion and communication

Each team completed these scales every 2 months as part of their self-assessment package (Appendix 2). Data are reported descriptively for months 1, 7, and 12 of the pilot.

Workgroup cohesion is the “degree to which an individual believes that the members of his or her work group are attracted to each other, are willing to work together, and are committed to completing the tasks and goals of the work group” [45]. We measured work cohesion using 8 items proposed by Riordan and Weatherly (1999). Based on the results of a cognitive debriefing exercise conducted with TREC care aides, the wording of each item was modified slightly to meet the project context, using appropriate language without losing meaning (e.g., revising the original statement ‘In my work group, there is a lot of team spirit among members’, to ‘we have a lot of team spirit among members’). Each scale item was scored on a seven-point Likert scale ranging from ‘strongly disagree’ to ‘strongly agree’. Item responses were averaged to provide an overall score ranging from 1 to 7; the latter score represents strong agreement about team cohesion, while a score of ‘4’ equals a ‘neutral’ response.

Workgroup communication is the “degree to which information is transmitted among the members of the work group” [45]. This construct was measured using 4 items, also adapted to align with the pilot (e.g., changing the original statement ‘In my work group, individuals frequently discuss work assignments with each other’ to ‘We frequently discuss resident care assignments with each other’). Scoring occurred as per workgroup cohesion. Cronbach’s alpha was high for the original versions of the workgroup cohesion (∝ = .92) and workgroup communication (∝ = .79) scales [45].

### Resident quality indicators

Quality indicators were assessed using RAI-MDS 2.0 data [46]. The standard for reporting these data in Canada is set by the Canadian Institute of Health Information (https://www.cihi.ca/en/about-cihi). Throughout most of Canada, all nursing home residents are required to have a full-length assessment completed close to their time of admission and annually thereafter, interspersed by abbreviated quarterly assessments. Full-length assessments contain about 400 standardized items that are completed by a trained assessor (usually a nurse) using data from clinical charts and direct observations. These data are used to profile nursing home residents (e.g., by their cognitive and functional performance) and to provide indicators of quality care (e.g., the percent of residents with improved mobility or worsening pain).

We obtained assessment-level RAI-MDS 2.0 data for each of the SCOPE units. Data were obtained for a 3-year period, starting 2 years before SCOPE and ending at the completion of the pilot (i.e., January 2014 to March 2017). Specific to the clinical area chosen by teams, we assessed unit-level changes in the percentage of residents who showed improvements in mobility, whose responsive behavioural symptoms improved, or with pain. Resident mobility was assessed using the third generation [47] RAI-MDS 2.0 quality indicator “MOB1a” (the percentage of residents whose ability to locomote on the unit improved). This indicator excludes residents who are comatose, have six or fewer months to live, and/or who were independently mobile during their previous RAI-MDS 2.0 assessment [46]. The quality indicator entitled “BEHI4” was used to identify the percentage of residents on each unit whose behavioral symptoms (i.e., wandering, verbally abusive, physically abusive, socially inappropriate or disruptive behavior) improved from the previous RAI-MDS 2.0 assessment [46]. This indicator excludes residents who are comatose or who had missing behavioral scores in their previous assessment. Resident pain was measured using the RAI-MDS 2.0 pain scale [46]. This quality indicator assesses the percentage of residents with any amount of pain in the last seven days, excluding those with missing or conflicting (no pain frequency but with some degree of intensity) item responses.

Unit-level changes in RAI-MDS 2.0 quality indicators are presented using statistical process control (SPC) charts [48]. Data were not normally distributed and thus the following SPC zones were created using pre-SCOPE (January, 2013 to December 2016) data: (a) zone ^−^3 = 1st–5th percentile; (b) zone ^−^2 = 5th–34th percentile; (c) zone ^−^1 = 34th-50th percentile; (d) zone ^+^1 = 50th–66th percentile; (e) zone ^+^2 = 66th–95th percentile; (f) zone ^+^3 = 95th–99th percentile. SPC charts allow us to assess changes in processes or outcomes with time, and assume that in ‘null effect’ scenarios, data will be randomly distributed around a measure of central tendency [48]. Following the SPC Western Electric rules [49], non-random variation was detected if (a) one or more data points during the SCOPE pilot were beyond zone 3 of pre-SCOPE results, (b) two of three successive data points were beyond zone 2, or (c) four of five successive data points were beyond zone 1.

## Results

### Nursing home characteristics, team composition, and QI focus

The characteristics of SCOPE nursing homes, units and team composition are found in Table 2. Five of the 7 nursing homes in the pilot were (public or voluntary) non-profit, while 2 and 4 homes were medium (80–120 beds) and large (> 120 beds), respectively. Homes had between 1 and 6 units that ranged in size from 27 to 100 beds.

**Table 2:** SCOPE nursing home and unit characteristics

Site	Owner-operator type	Facility size^a^	# of units/facility	Unit size (# of beds)
A	Voluntary not for profit	Large	4	40
B	Voluntary not for profit	Large	5	27
C	Private for profit	Large	4	100
D	Public not for profit	Medium	2	40
E	Private for profit	Large	6	31
F	Voluntary not for profit	Small	1	57
G	Voluntary not for profit	Medium	4	29

^a^ Small (< 80 beds), medium (80–120 beds), large (> 120 beds)

Five of the seven SCOPE teams focused on reducing dementia-related responsive behaviors, 1 team focused on reducing pain, and 1 focused on improving resident mobility (Table 3). Team and senior sponsors were most often clinical nurse managers and Directors of Care, respectively. Team size, including the team and senior sponsor, ranged from 5 (*n* = 4 SCOPE sites) to 7 (*n* = 1 SCOPE site) individuals. With two exceptions (sites C and F), front-line SCOPE teams were comprised entirely of care aides.

**Table 3:** Team composition and quality improvement topic

Site	Quality improvement topic	Team composition					
		Care aides	Nurses	Other staff	Team sponsor	Senior sponsor	Total team size
A	Responsive behavior	4	0	0	Unit manager	DOC	*N* = 6
B	Responsive behavior	3	0	0	DOC	CEO	*N* = 5
C	Responsive behavior	3	1	0	Unit manager	DOC	*N* = 6
D	Responsive behavior	3	0	0	Registered nurse	DOC	*N* = 5
E	Responsive behavior	3	0	1 rec therapy 1 social worker	Unit manager	DOC	*N* = 7
F	Pain	3	0	0	Unit manager	DOC	*N* = 5
G	Mobility	3	0	0	Unit manager	DOC	*N* = 5

*Acronyms*: *CEO* chief executive officer, *DOC* director of care

### Treatment enactment

We rated 3 of the 7 teams as creating excellent aim statements (rating of 5/5) during the pilot (Table 4), 2 teams as creating adequate aim statements (rating = 3/5), and 2 teams as creating ‘poor’ (rating = 1/5), or ‘poor-to adequate’ (rating = 2/5) aim statements. To illustrate, team D was rated as having an excellent AIM statement. This team defined responsive behavior in their aim statement (‘hitting, screaming, arguing’; *specific*), quantified their goals (reducing events by 60%; *measurable*), showed progression throughout the pilot (reducing events by 60% at month 7, and 90% by month 12; *achievable and timely*), and defined when the intervention would occur (during activities of daily living; *relevant*) (data not shown). While Team B (rated as adequate) satisfied the ‘specific’ (defined responsive behavior), ‘measurable’ (included a target goal) and ‘relevant’ (reported when the intervention would occur) SMART criteria, this team did not show progression in its aim statement, and nor did it identify a timeline for achieving intervention success. We rated team E as having a poor aim statement, as it met the ‘relevant’ SMART criteria only (defined when the intervention would occur).

**Table 4:** Ratings of treatment enactment during the SCOPE pilot

Site	Quality improvement topic	AIM statements rating	Intervention progression rating	Use of measurement to guide decisions rating
A	Responsive behavior	5	4	3
B	Responsive behavior	3	1	3
C	Responsive behavior	3	3	1
D	Responsive behavior	5	3	5
E	Responsive behavior	1	1	3
F	Pain	2	5	5
G	Mobility	5	5	5

Scoring: *1* poor, *2* poor to adequate, *3* adequate, *4* adequate to excellent, *5* excellent

Each team was also rated on their intervention progress. We rated 5 teams as achieving adequate to excellent intervention progression (Table 4); however, only Teams F and G reported scaling their intervention to involve other residents and/or staff on their unit (these teams received a rating of ‘excellent’). Team F reported using ‘pain pocket card survival kits’ to remind and help unit staff to implement the intervention, and reflected on how they engaged with non-SCOPE providers on their unit to enhance their care processes. We rated teams B and E as achieving poor intervention progression; both teams reported a ‘success story’ for only one resident at the end of the pilot (data not shown).

Teams D, F, and G specifically reported how they used measurement tools (e.g., mobility tracking tools, use of RAI-MDS 2.0 data) to help make decisions throughout the pilot, and hence we rated these teams as ‘excellent’ in this category (Table 4). Teams A, B, and E vaguely referred to measurement (e.g., conducting baseline assessments) without providing details, and were rated as ‘adequate’. Team C did not make any reference to using measurement to guide decisions.

### Workgroup cohesion and communication

Team cohesion and communication results are shown in Table 5. Most teams moderately (an average score of ‘6’ across all scale questions) or strongly (an average score of ‘7’ across all questions) agreed with statements about their cohesion and communication throughout the pilot. As the only exception, team C provided a score of 3.8 (a neutral opinion) for team cohesion at month 12 of the pilot.

**Table 5:** Self-reported measures of workgroup cohesion and communication during the pilot

Site	Quality improvement topic	Workgroup cohesion^a^			Workgroup communication^a^		
		Month 1^c^	Month 7	Month 12	Month 1	Month 7	Month 12
A	Responsive behavior	5.8	6.9	5.8	6.0	7.0	6.0
B	Responsive behavior	5.6	6.4 ^b^	5.9	6.5	7.0^c^	6.0
C	Responsive behavior	7.0	6.0	3.8	7.0	6.0	Not completed
D	Responsive behavior	7.0	6.0	6.3	7.0	7.0	Not completed
E	Responsive behavior	4.3	Not completed	6.0	4.5	Not completed	6.0
F	Pain	6.0	6.6	6.3	6.0	7.0	7.0
G	Mobility	7.0	6.0	7.0	7.0	6.5	7.0

^a^ One score provided per team

^b^ Data were missing for month 7, and were replaced by month 9 (October, 2016) team responses

^c^ *Month 1* February, 2016, *Month 7* August, 2016, *Month 12* February, 2017

### Resident quality indicators

**Fig. 2:**
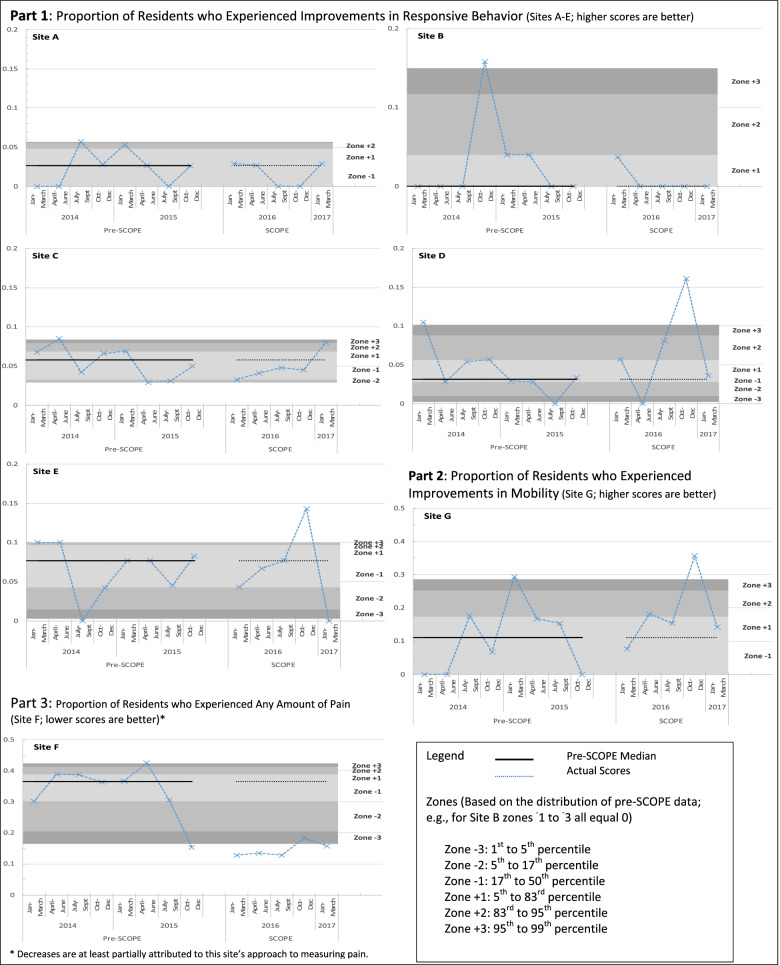
Unit-level clinical outcomes prior to and during the SCOPE pilot

SPC charts for quality indicators are shown in Fig. 2. Patterns of quality care indicator data were non-random for sites D and E (responsive behaviors; one data point beyond zone ^+^3), showing improvements in responsive behaviours for two of the five sites that worked on this clinical area during the SCOPE pilot. SPC results also show improvements for Site G that worked on improving mobility (this site had one data point beyond zone ^+^3 towards the end of the SCOPE pilot), and site F that worked on improving pain (this site had 4 consecutive data points beyond zone ^−^1 during the SCOPE pilot). We noted that the improvement pattern for site F commenced pre-SCOPE. During follow-up discussions, site F leaders disclosed that this was at least partly due to changes in their pain assessment approach (i.e., all residents receiving an analgesic were originally deemed as having pain). Three of the 4 teams (sites D, F, G) who showed improved quality care also received a score of ‘excellent’ in at least 2 of the treatment enactment areas reported in Table 4, versus only 1 of 3 teams with random changes in quality care (while Site A was rated as creating an excellent aim statement, changes in responsive behavior for this unit were coded as random).

## Discussion

This pilot demonstrated that the bundle of SCOPE teaching and coaching strategies—a “getting started” information kit, structured learning sessions, quality advisor guidance, and discussions to help senior and team sponsors support front-line teams—effectively supported most care aide-led teams’ to enact their QI strategies. Five of the seven teams provided adequate or excellent aim statements during the pilot, five teams reported achieving at least adequate intervention progress (i.e., showed learning and refinement through PDSA cycles), and three teams specifically discussed how they used measurement to guide intervention decision making processes during the pilot. Teams that scored high in one enactment area tended to do so in others (e.g., site G received a score of ‘excellent’ in each of the enactment areas, sites D and F received this score in two of three areas), while conversely, teams that scored poorly in one enactment area tended to consistently do so (see site E in Table 4). As discussed by Kilo (1998), this pattern of results emphasizes the need for PDSA training to reflect the interconnected nature of AIM development, care plan implementation, and measurement [38].

Additionally, while this pilot was not powered to detect statistically significant differences in measures of treatment efficacy, it is important to note that (1) some trends for improvement in quality indicators were noted at the resident care-unit level, and (2) these trends occurred more often amongst teams who successfully enacted SCOPE. These results help demonstrate that successfully facilitated coaching strategies have the potential to impact resident care, which hence provides support to further adapt and refine the SCOPE intervention in future studies.

Our pilot study contributes to existing nursing home quality improvement and implementation research [50, 51] in three ways. First, our results contribute to the growing body of literature showing that care aides can successfully lead QI initiatives, with the proper support. This is important, given care aides’ essential role in providing day-to-day nursing home support coupled with their high degree of knowledge about the wants and needs of residents [22–24]. Actively engaging with care aides is important to enhance nursing home quality of care, particularly given the need to balance effective medical care with relational and social approaches [12, 13]. Several researchers have demonstrated the benefits of meaningfully engaging both care staff [28–30] and residents [52, 53] during care processes.

Second, these findings contribute to our understanding of how facilitated coaching can help to support quality improvement interventions. As proposed by Rycroft-Malone and colleagues [40, 54], our pilot results suggest that a combination of technical (e.g., Getting Started kits that provide teams with important background clinical information and examples of evidence-informed interventions), educational (e.g., structured learning sessions that show teams how to apply PDSA models ), and ongoing facilitated coaching strategies (e.g., quality advisors that help teams to integrate their care plans into daily care activities and to overcome barriers as they arise) are all required to support complex QI interventions. These findings are complemented by an earlier qualitative study conducted by Ginsburg et al. (2018) who analyzed data from 6 focus groups conducted during our final SCOPE celebratory conference [41]. While care aides in this study felt that all components of SCOPE were important, they also reported considerable challenges with measurement, recommended less didactic teaching, and asked us to include more pragmatic examples of measurement tools in the Getting Started kits.

Third, these study findings highlight the need to develop more detailed process evaluation techniques that allows us to better understand both how and why interventions succeed or fail [55]. While intervention fidelity is traditionally measured using self-report strategies [42], these data are prone to information bias [56], and techniques are required to differentiate between what an intervention has taught (fidelity delivery), what is learned (fidelity receipt), and what is implemented by teams (fidelity enactment). Enactment was measured from one data source in the present study (researcher coding of care aide self-reports). Future studies would benefit from using a range of data sources and methods, including care aide and sponsor self-reports and external assessments (e.g., quality advisor diaries and/or researcher observations). Our research team continues to refine the enactment rubric used in this pilot, both to expand the tool measurement domains (e.g., differentiating between treatment receipt and enactment) and to create more refined scoring criteria, for use in future endeavours.

## Limitations

SCOPE teams were recruited from a single Canadian health region, and hence lessons learned should be applied cautiously to other jurisdictions and countries. We also did not investigate how site leaders selected SCOPE team members and their intervention foci, which may have influenced study outcomes. Teams provided self-reported scores of treatment enactment without explaining how these assessments were decided (e.g., by team consensus, by one person on behalf of the team). More detailed and objective approaches to assessing fidelity enactment will help to provide more robust data on this important construct. Similarly, data on team cohesion and communication showed potential ceiling effects; these data were self-reported at the team level and social desirability or selection bias may explain the high scores on these measures. In future research, individual team-member responses may provide more accurate data. Alternative measures of team dynamics should also be explored and considered for use, and/or qualitative methods of inquiry could be used, to more richly assess that ways in which team dynamics influences intervention enactment.

## Conclusion

This pilot provides knowledge to guide future care aide-led nursing home quality improvement initiatives by (1) showing that care aides can effectively lead QI initiatives; (2) illustrating that PDSA training to support these initiatives should emphasize the interconnected nature of AIM development, care plan implementation, and measurement; (3) providing insights into the ways in which SCOPE could be modified in future research, and; 4) developing and implementing a rubric to assess fidelity enactment. Modifications to this tool are required to measure additional enactment domains (fidelity receipt) while incorporating different sources of data.

## Supplementary Information

**Additional file 1: Appendix 1.** This file provides the daily agendas for each SCOPE Learning Session.

**Additional file 2: Appendix 2.** This file shows the SCOPE Quality Improvement Team Assessment Form, completed bi-monthly during pilot. Responses to this form were used to score team enactment.

## Data Availability

The data that support the findings of this study are available from Translating Research in Elder Care, c/o Dr. Carole Estabrooks, but restrictions apply to the availability of these data, which were used under license for the current study, and so are not publicly available. Data are however available from the authors upon reasonable request and with permission of Dr. Carole Estabrooks.

## Abbreviations

QI: Quality improvement
SCOPE: Safer Care for Older Persons in Residential Care Environments
TREC: Translating Research in Elder Care
PDSA: Plan-do-study-act
IHI: Institute for Healthcare Improvement
SPC: Statistical process control
