# Correction to: SCOPE: safer care for older persons (in residential) environments—a pilot study to enhance care aide-led quality improvement in nursing homes

**DOI:** 10.1186/s40814-022-01004-4

**Published:** 2022-03-01

**Authors:** Malcolm Doupe, Thekla Brunkert, Adrian Wagg, Liane Ginsburg, Peter Norton, Whitney Berta, Jennifer Knopp-Sihota, Carole Estabrooks

**Affiliations:** 1 Max Rady College of Medicine, Rady Faculty of Health Sciences, University of Manitoba, Winnipeg, MB Canada; 2 University Department of Geriatric Medicine FELIX PLATTER, Basel, Switzerland; 3 Nursing Science (INS), Department Public Health (DPH), Faculty of Medicine, University of Basel, Basel, Switzerland; 4 Department of Medicine, Faculty of Medicine and Dentistry, University of Alberta, Edmonton, Alberta Canada; 5 School of Health Policy & Management, York University, Toronto, Canada; 6 Department of Family Medicine, University of Calgary, Calgary, Alberta Canada; 7 Institute of Health Policy, Management & Evaluation, University of Toronto, Toronto, Canada; 8 Faculty of Health Disciplines, Athabasca University, Edmonton, Alberta Canada; 9 Faculty of Nursing, University of Alberta, Edmonton, Alberta Canada

**Correction to: Pilot Feasibility Stud 8, 26 (2022)**

**https://doi.org/10.1186/s40814-022-00975-8**

Following publication of the original article [1], the authors reported an error in Fig. 2. The correct figure is given below.

**Fig. 2:**
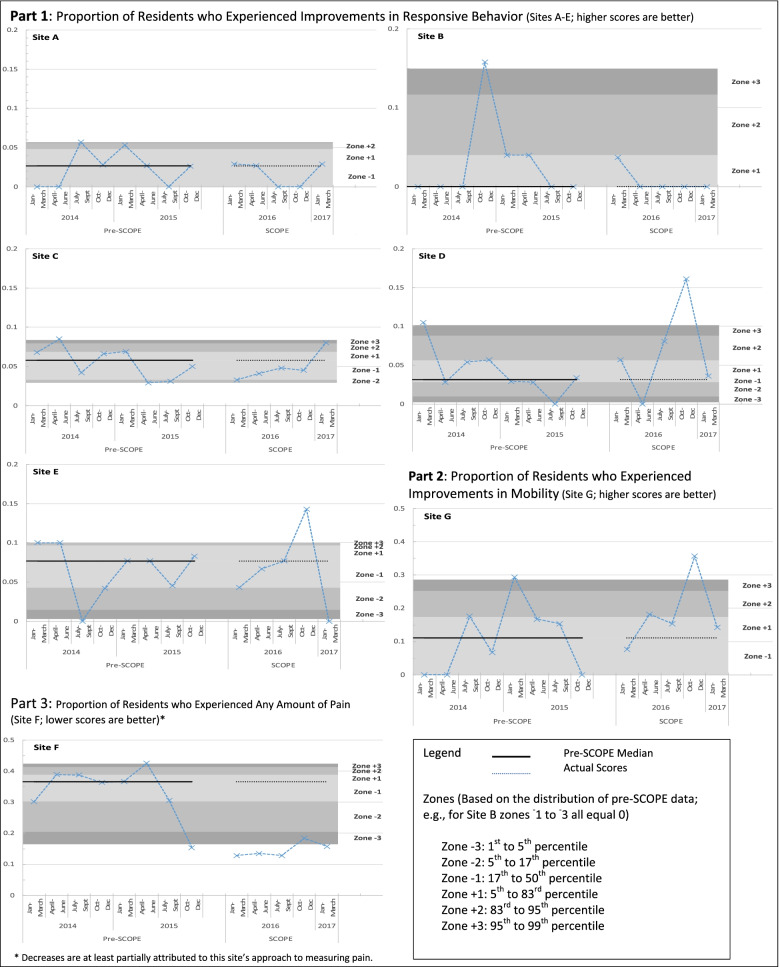
Unit-level clinical outcomes prior to and during the SCOPE pilot

The original article [1] has been updated.
