# Supplementary material for: SCOPE: safer care for older persons (in residential) environments—a pilot study to enhance care aide-led quality improvement in nursing homes
Source: Pilot Feasibility Stud. 2022 Feb 3;8:26. doi: 10.1186/s40814-022-00975-8 (PMC8812152; doi:10.1186/s40814-022-00975-8)
Supplement: Supplementary file 1 — Additional file 1: Appendix 1. This file provides the daily agendas for each SCOPE Learning Session. [file 40814_2022_975_MOESM1_ESM.docx]

**Safer Care for Older Persons (in Residential) Environments**

**Learning Congress 1**

Inn at The Forks, 75 Forks Market Rd, Winnipeg, MB R3C 0A2

**Day One: February 8, 2016**

| **Time** | **Topic^a^** |  |
| --- | --- | --- |
| 1030 - 1200 | Site Sponsor and Team Sponsor Session* |  |
| 1130 - 1230 | **Registration, Lunch and Storyboard Setup** | |
| 1230 - 1245 | Welcome *– Why are we here?* |  |
| 1245 - 1300 | Impromptu Networking *- Sharing challenges, expectations, building new connections* |  |
| 1300 - 1320 | What I experienced in the SCOPE pilot project |  |
| 1320 - 1325 | What does the day look like? |  |
| 1325 - 1400 | Storyboard Review *– Who we are? Teams circulate, review storyboards and ask questions* |  |
| 1400-1430 | **Stretch Break & Coffee** | |
| 1430 - 1515 | The Big Three:  Resident Pain, Behaviour and Mobility |  |
| 1515 - 1605 | Trying to get better – *How can we do it?* |  |
| 1605 - 1620 | Team Charter Review *– What do you want to work on?* |  |
| 1620 - 1630 | What was the best thing you heard today? |  |
| 1630 | The end of Day 1 |  |
| 1700 - 2030 | **Dinner and Evening Fun Event** | |

***** This session is intended for PCH Site Sponsor and Team Sponsor.
^a^ Presenters names have been removed to protect privacy

**Safer Care for Older Persons (in Residential) Environments**

**Learning Congress 1**

Inn at The Forks, 75 Forks Market Rd, Winnipeg, MB R3C 0A2

**Day Two: February 9, 2016**

| **Time** | **Presentation^a^** |
| --- | --- |
| 0800 – 0830 | **Buffet Breakfast** |
| 0830 – 0845 | Welcome to Day 2 *– Ready for more?* |
| 0845 - 0915 | What is an AIM statement? |
| 0915 - 1000 | Let’s Acti it Out *– Improv and Simulation* |
| 1000 – 1030 | **Stretch Break & Coffee** |
| 1030 – 1100 | What are your ideas for change? – Team breakout |
| 1100 - 1130 | How do you make sure your team is dysfunctional and not successful? |
| 1130 – 1200 | How do we know we are making a difference? |
| 1200 – 1230 | **Lunch** |
| 1230 – 1300 | Deeper Dive *– What can we try by next Tuesday?* |
| 1300 – 1340 | The Journey has Started!  *What needs to happen between now and Learning Congress 2?* |
| 1340 – 1400 | **Stretch Break** |
| 1400 – 1435 | Story Telling Eye Opener |
| 1435 – 1515 | Making Action Periods Successful |
| 1515 – 1530 | Participant Feedback |
| 1530 | End of Day 2 – Send off! |

^a^ Presenters names have been removed to protect privacy

**Safer Care for Older Persons (in Residential) Environments**

**Learning Congress 2**

Fort Gibraltar, 866 Rue Saint Joseph, Winnipeg, MB R2H 0G4

**Day One: May 30, 2016**

| **Time** | **Topic^a^** |  |
| --- | --- | --- |
| 0800 – 0830 | **Registration, Breakfast, Story Board Setup** | |
| 0830 – 0840 | Welcome Back |  |
| 0840 – 0850 | Impromptu Networking – Sharing challenges, expectations, building new connections |  |
| 0850 – 0930 | Our Stories – Team Presentations  Team 1  Team 2  Team 3  Team 4 |  |
| 0930 – 0945 | Biggest Challenge and Biggest Opportunity |  |
| 0945 – 1000 | **Stretch Break & Coffee** | |
| 1000 – 1030 | Our Stories – Team Presentations  Team 5  Team 6  Team 7 |  |
| 1030 – 1130 | Measurement and AIM Statement |  |
| 1130 – 1200 | **Lunch** |  |
| 1230 – 1255 | Staff Engagement |  |
| 1255 – 1310 | My Story |  |
| 1310 – 1345 | How do we know we are making a difference? |  |
| 1345 – 1350 | Wrap Up – Day 1 |  |
| 1350 – 1600 | Team Activity |  |

* Note: Session is intended for PCH Site Sponsor and Team Sponsor.

^a^ Presenters names and Teams have been removed to protect privacy

**Safer Care for Older Persons (in Residential) Environments**

**Learning Congress 2**

Fort Gibraltar, 866 Rue Saint Joseph, Winnipeg, MB R2H 0G4

**Day Two: May 31, 2016**

| **Time** | **Presentation^a^** |  |
| --- | --- | --- |
| 0800 – 0830 | **Breakfast** | |
| 0830 – 0840 | Welcome to Day 2 *– Ready for more?* |  |
| 0840 – 1040 | Leadership Session (Site Sponsors & Team Sponsors) |  |
| 0840 – 0910 | Finding Gems |  |
| 0910 – 0940 | Thinking Differently |  |
| 0940 – 1010 | Ideas that get you closer to your goal |  |
| 1010 – 1040 | Beyond the Driver Diagram |  |
| 1040 – 1055 | **Stretch Break & Coffee** | |
| 1055 – 1125 | Beyond Low Hanging Fruit |  |
| 1125 – 1200 | Change Packages – What ideas can be added |  |
| 1200 – 1230 | **Lunch** |  |
| 1230 – 1250 | Seniors Leaders Report Out |  |
| 1250 – 1330 | Team Meeting – What do we need to work on? |  |
| 1330 – 1400 | Best ideas from Learning Congress 2 |  |
| 1400 – 1445 | Learning Congress 2 – Wrap up and Evaluation |  |

^a^ Presenters names have been removed to protect privacy

**Safer Care for Older Persons (in Residential) Environments**

**Learning Congress 3**

Manitoba Legislative Building, 450 Broadway, Winnipeg, MB R3C 0V8

**Day One: October 17, 2016**

| **Time** | **Topic^a^** |  |
| --- | --- | --- |
| 0800 – 0830 | **Registration, Breakfast, Story Board Setup** | |
| 0830 – 0840 | Welcome Back |  |
| 0840 – 0910 | Common Group and Reflections |  |
| 0910 – 0930 | Our Stories – Team Presentations  Team 5  Team 3 |  |
| 0930 – 1030 | Clinical Change Ideas – Session 1 |  |
| 1030 – 1045 | **Stretch Break & Coffee** | |
| 1045 – 1110 | Our stories – Team Presentations  Team 2  Team 1 |  |
| 1110 – 1125 | Reflections from the SCOPE Pilot Project |  |
| 1125 – 1145 | Getting Unstuck |  |
| 1145 – 1215 | **Lunch** |  |
| 1215 – 1250 | Our Stories  Team 6  Team 4  Team 7 |  |
| 1250 – 1340 | Clinical Change Ideas – Session 2 |  |
| 1340 – 1430 | Team Planning and Wrap Up |  |
| 1430 – 1530 | Team Activity |  |

^a^ Presenters names and Teams have been removed to protect privacy

**Safer Care for Older Persons (in Residential) Environments**

**Learning Congress 3**

Manitoba Legislative Building, 450 Broadway, Winnipeg, MB R3C 0V8

**Day Two: October 18, 2016**

| **Time** | **Presentation^a^** |  |
| --- | --- | --- |
| 0800 – 0830 | **Breakfast** | |
| 0830 – 0840 | Welcome to Day 2 – *Ready for more?* |  |
| 0840 – 0925 | How do we know an improvement has occurred |  |
| 0925 – 0955 | Spreading Change |  |
| 0955 – 1015 | Spreading Change – BC Pilot Experience |  |
| 1015 – 1030 | **Stretch Break & Coffee** | |
| 1030 – 1100 | Sustaining Great Work |  |
| 1100 – 1115 | Perspectives |  |
| 1115 – 1200 | Improvement Clinic |  |
| 1200 – 1320 | **Lunch – Leadership Session* (Site Sponsors & Team Sponsors)** |  |
| 1220 – 1230 | **Lunch - Teams** |  |
| 1230 – 1320 | Resilience |  |
| 1320 – 1405 | Team Planning for Action Period 3 |  |
| 1405 – 1410 | Evaluation |  |
| 1410 – 1430 | Closing Remarks and Send Off |  |

* Note: Session is intended for PCH Site Sponsor and Team Sponsor.

^a^ Presenters names have been removed to protect privacy

**Safer Care for Older Persons (in Residential) Environments**

**Celebration Congress**

Forks Ballroom, Inn at The Forks, 75 Forks Market Rd.

**February 10, 2017**

| **Time** | **Topic^a^** |  |
| --- | --- | --- |
| 0830 – 0900 | **Registration, Breakfast, Story Board Setup** | |
| 0900 – 0915 | Welcome |  |
| 0915 – 0930 | Lights, Camera, Action! |  |
| 0930 – 0950 | Story Board Review |  |
| 0950 – 1010 | Team 7 |  |
| 1010 – 1030 | Team 6 |  |
| 1030 – 1050 | Team 2 |  |
| 1050 – 1105 | **Stretch Break & Coffee** | |
| 1105 – 1125 | Team 5 |  |
| 1125 – 1145 | Team 3 |  |
| 1145 – 1205 | Team 4 |  |
| 1205 – 1225 | Team 1 |  |
| 1225 – 1255 | **Lunch** |  |
| 1255 – 1355 | Focus Groups |  |
| 1355 – 1425 | A Picture is Worth a Thousand Words |  |
| 1425 – 1445 | What We Learned and What’s Next |  |
| 1445 – 1520 | Team Recognition |  |
| 1520 – 1530 | Final Remarks & Evaluation |  |

^a^ Presenters names and Teams have been removed to protect privacy
