# Supplementary material for: SCOPE: safer care for older persons (in residential) environments—a pilot study to enhance care aide-led quality improvement in nursing homes
Source: Pilot Feasibility Stud. 2022 Feb 3;8:26. doi: 10.1186/s40814-022-00975-8 (PMC8812152; doi:10.1186/s40814-022-00975-8)
Supplement: Supplementary file 2 — Additional file 2: Appendix 2. This file shows the SCOPE Quality Improvement Team Assessment Form, completed bi-monthly during pilot. Responses to this form were used to score team enactment. [file 40814_2022_975_MOESM2_ESM.docx]

**Appendix 2**

**SCOPE Quality Improvement Team Assessment Worksheet**

**and**

**Scales Used to Measure Workgroup Cohesion and Communication**

Aims of the Team:

Date:

Site/Location:

Facility & Team Name:

*Report for:*

Changes tested through PDSA cycles this past month:
What we learned from Tests of Change this month:
Main Accomplishments this past month:
**Describe Measures** - (Attach Annotated Run Charts)

**Process measures** (are we doing what we said we would do?)

**Outcome measures** (are things getting better?)

**Balancing measures** (are we making other areas/outcomes worse with this test?)

**Team Self-Assessment:**

***What this means:***

**1 - Team formed**

Team formed. Aim determined. Some team members attended Learning Session 1

**2 - Activity but no testing**

Team engaged in developing changes. No tests of change (PDSA) within last month

**3 - Changes tested, but no improvement**

Testing (PDSA) has begun, but there is no evidence of improvement

**4 - Changes tested, some improvement**

Team is putting change approaches into use on unit. Improvements have reached 50% of at least one goal

**5 - Significant Improvement**

100% of at least one goal is reached

**6 - Outstanding Sustainable Results**

Targets exceeded. Changes spread to other units

Place a single X on the scale that best represents your team’s progress in achieving your aims based on group consensus.

________________________________________________________________________

1 2 3 4 5 6

Significant

Improvement

Changes tested, some improvement

Changes tested, but no improvement

Activity but no testing

Team formed

Outstanding sustainable results

**Intra-team relationships (Work Group Cohesiveness Scale):**
Please indicate your level of agreement with each of the following statements about your work group.

|  | Level of Agreement | | | | | | |
| --- | --- | --- | --- | --- | --- | --- | --- |
|  | Strongly Disagree | Moderately  Disagree | Slightly Disagree | Neutral | Slightly  Agree | Moderately  Agree | Strongly  Agree |
| 1. We have a lot of team spirit among members. | ❑ 1 | ❑ 2 | ❑ 3 | ❑ 4 | ❑ 5 | ❑ 6 | ❑ 7 |
| 2. We know that we can depend on each other. | ❑ 1 | ❑ 2 | ❑ 3 | ❑ 4 | ❑ 5 | ❑ 6 | ❑ 7 |
| 3. We stand up for each other. | ❑ 1 | ❑ 2 | ❑ 3 | ❑ 4 | ❑ 5 | ❑ 6 | ❑ 7 |
| 4. We pitch in to help each other. | ❑ 1 | ❑ 2 | ❑ 3 | ❑ 4 | ❑ 5 | ❑ 6 | ❑ 7 |
| 5. We take interest in one another. | ❑ 1 | ❑ 2 | ❑ 3 | ❑ 4 | ❑ 5 | ❑ 6 | ❑ 7 |
| 6. We regard each other as friends. | ❑ 1 | ❑ 2 | ❑ 3 | ❑ 4 | ❑ 5 | ❑ 6 | ❑ 7 |
| 7. We are very cooperative with one another. | ❑ 1 | ❑ 2 | ❑ 3 | ❑ 4 | ❑ 5 | ❑ 6 | ❑ 7 |
| 8. We work as an effective team. | ❑ 1 | ❑ 2 | ❑ 3 | ❑ 4 | ❑ 5 | ❑ 6 | ❑ 7 |

**Intra-team relationships (Work Group Communication Scale):** Communication within our own team
Please indicate your level of agreement with each of the following statements about your work group.

|  | Level of Agreement | | | | | | |
| --- | --- | --- | --- | --- | --- | --- | --- |
|  | Strongly Disagree | Moderately  Disagree | Slightly Disagree | Neutral | Slightly  Agree | Moderately  Agree | Strongly  Agree |
| 1. We feel free to offer an opinion regarding work-related issues. | ❑ 1 | ❑ 2 | ❑ 3 | ❑ 4 | ❑ 5 | ❑ 6 | ❑ 7 |
| 2. We frequently discuss resident care assignments with each other. | ❑ 1 | ❑ 2 | ❑ 3 | ❑ 4 | ❑ 5 | ❑ 6 | ❑ 7 |
| 3. We can share ideas and information. | ❑ 1 | ❑ 2 | ❑ 3 | ❑ 4 | ❑ 5 | ❑ 6 | ❑ 7 |
| 4. We take the time to listen to coworkers’ problems and worries. | ❑ 1 | ❑ 2 | ❑ 3 | ❑ 4 | ❑ 5 | ❑ 6 | ❑ 7 |
